# Lung Cancer Screening with Low-Dose CT in Smokers: A Systematic Review and Meta-Analysis

**DOI:** 10.3390/diagnostics11061040

**Published:** 2021-06-05

**Authors:** Theresa Hunger, Eva Wanka-Pail, Gunnar Brix, Jürgen Griebel

**Affiliations:** Department of Medical and Occupational Radiation Protection, Federal Office for Radiation Protection, Ingolstaedter Landstrasse 1, 85764 Oberschleissheim, Germany; ewanka-pail@bfs.de (E.W.-P.); gbrix@bfs.de (G.B.); juergen-griebel@gmx.de (J.G.)

**Keywords:** screening, lung cancer, low-dose CT, systematic review

## Abstract

Lung cancer continues to be one of the main causes of cancer death in Europe. Low-dose computed tomography (LDCT) has shown high potential for screening of lung cancer in smokers, most recently in two European trials. The aim of this review was to assess lung cancer screening of smokers by LDCT with respect to clinical effectiveness, radiological procedures, quality of life, and changes in smoking behavior. We searched electronic databases in April 2020 for publications of randomized controlled trials (RCT) reporting on lung cancer and overall mortality, lung cancer morbidity, and harms of LDCT screening. A meta-analysis was performed to estimate effects on mortality. Forty-three publications on 10 RCTs were included. The meta-analysis of eight studies showed a statistically significant relative reduction of lung cancer mortality of 12% in the screening group (risk ratio = 0.88; 95% CI: 0.79–0.97). Between 4% and 24% of screening-LDCT scans were classified as positive, and 84–96% of them turned out to be false positive. The risk of overdiagnosis was estimated between 19% and 69% of diagnosed lung cancers. Lung cancer screening can reduce disease-specific mortality in (former) smokers when stringent requirements and quality standards for performance are met.

## 1. Introduction

Early detection of diseases before they cause symptoms or discomfort is becoming increasingly important in the health care systems of many countries. The rationale is to diagnose diseases at such an early stage that an effective and less burdensome therapy becomes possible.

The rapid technological development of radiological imaging procedures in the recent years has not only led to an increasingly frequent use of these procedures in symptomatic persons, i.e., patients, but also to early diagnosis of asymptomatic persons [1]. This is particularly true for computed tomography (CT), which is predestined for screening of serious diseases due to its high spatial and temporal resolution.

However, the possible advantages of screening examinations must be carefully weighed against undesirable effects, such as false-positive and false-negative findings, invasiveness of the diagnostic work-up, and overdiagnosis [2]. In the case of radiological procedures, the radiation exposure and the resulting radiation risks of screening participants must also be taken into account. In this context, it is important to note that when screening a large population of asymptomatic persons, the vast majority of them will not have a direct benefit due to the low prevalence of the considered diseases, but all are subject to the aforementioned undesirable effects and radiation risks. Therefore, the International Basic Safety Standards for Protection against Ionizing Radiation [3] and the European Directive 2013/59/Euratom [4] have published basic conditions for the use of radiological imaging procedures in early detection. Both regulations place high demands on the justification process, i.e., the risk-benefit assessment, on the basis of scientific evidence. In Germany, these requirements are anchored in the Radiation Protection Law that requires approval of every screening procedure on the basis of a scientific evaluation. For this purpose, we assessed benefits and harms of lung cancer screening with low-dose computed tomography (LDCT) in smokers and former smokers.

In the European Union (EU), lung cancer is the leading cause of cancer death in males (European age standardized mortality rate in 2012, 66.3/100,000) and the second most common in females (20.6/100,000) after breast cancer [5]. By far the most significant risk factor is cigarette smoking. One in ten smokers develops lung cancer on average 30 to 40 years after starting to smoke [6]. The overall five-year survival rate in EU countries is 15% on average [7] and is strongly dependent on histology and stage at diagnosis. Therefore, the current focus of CT screening is on lung cancer in heavy smokers. In some countries, such as the U.S. or Canada, CT screening for lung cancer is already recommended and reimbursed [8,9]; in the EU, various medical professional societies and experts are advocating the introduction as an organized screening program [10,11].

It was thus the aim of the present review (i) to perform a systematic review of randomized controlled trials (RCT) of lung cancer screening with LDCT in order to estimate the benefit and undesirable effects of the screening approach and (ii) to provide an overview on the radiological procedures used in these studies.

## 2. Materials and Methods

We performed a systematic review in compliance with the Preferred Reporting Items for Systematic Reviews and Meta-Analyses (PRISMA) statement [12]. A search in electronic databases (Medline, Embase, Cochrane CENTRAL) was conducted with thesaurus and free-text terms for lung cancer, population screening, and computed tomography. The search was performed in April 2020. We also hand-searched bibliographies of eligible publications.

Full publications of studies were included if they were randomized controlled trials (RCTs) or systematic reviews of RCTs that compared CT screening with no screening or screening with chest radiogram (CXR) in current or former smokers. Studies should report results on benefits and/or harms of LDCT screening and cover the whole screening process from participant selection to LDCT screening, lung cancer diagnosis, and follow-up. The main outcome for this review was disease specific and overall mortality. Other outcomes of interest were lung cancer incidence, including information on stage and histology, radiation exposure, invasive diagnostic procedures, false-positive screening results, overdiagnosis, and health-related quality of life, including psychosocial consequences. The effect of screening on smoking behavior was investigated in an ad hoc analysis. No limits were set for publication date, but language was restricted to English and German.

Studies were selected by two independent reviewers with epidemiological backgrounds (T.H., E.W.-P.) in a two-step approach. First, all citations from the database search were screened on the basis of title and abstract. In the second step, relevant publications were selected according to the inclusion and exclusion criteria by reviewing the full text. The methodological quality of studies and their potential for bias regarding the primary endpoint was assessed with the Cochrane risk-of-bias tool [13]. Data on study characteristics, intervention details, and outcome data were extracted in standardized forms and double-checked. For the changes in smoking behavior, additional relevant articles from the database search were considered if they concerned the included RCT.

To compare the outcomes between screening and control arms across all studies, a meta-analysis with a random-effects model was performed for lung cancer specific and overall mortality with the Cochrane Review Manager 5.3 software (Copenhagen: The Nordic Cochrane Centre, The Cochrane Collaboration, 2014). Heterogeneity between studies was assessed with the I^2^ statistics. Studies were grouped according to mode of control, i.e., no screening versus screening with CXR, and results stratified for gender were analyzed in subgroups. Sensitivity analyses were performed with restriction to high quality studies and to studies with comparable mode of control, respectively. Results for other outcomes, including smoking behavior, as well as radiological aspects, were summarized narratively and in evidence tables. Risk of overdiagnosis was calculated as the difference in lung cancer incidence between screening and control group [14].

## 3. Results

### 3.1. Search Results

**Figure 1:**
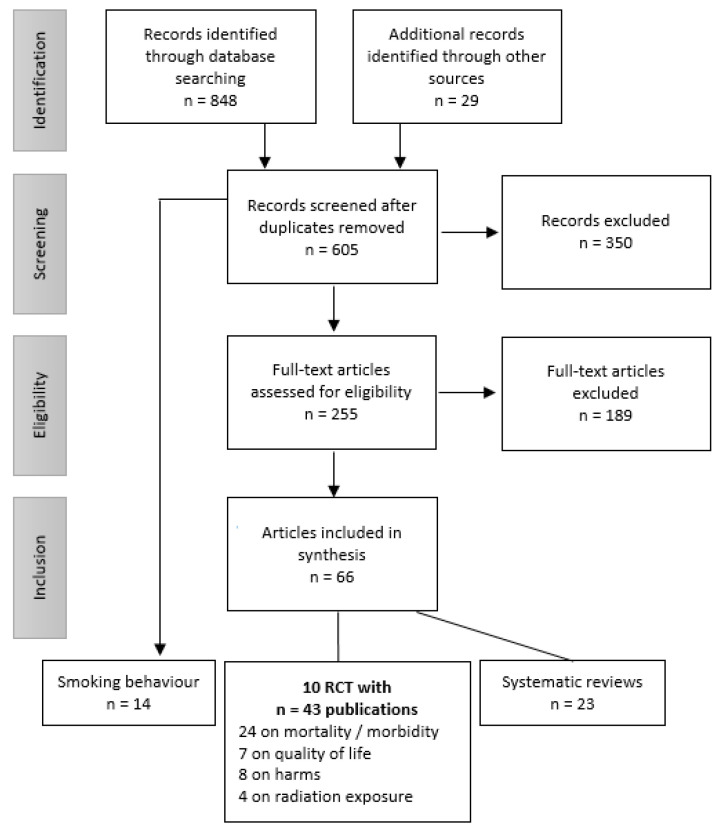
Flow chart of literature selection.

The electronic database search yielded 848 citations; the by-hand search added another 29 references (Figure 1). After removing duplicates, 605 citations were screened for eligibility on the basis of title and abstract, and 255 publications were evaluated in full text. After application of the inclusion and exclusion criteria, 43 publications of ten RCTs (DANTE, Depiscan, DLCST, ITALUNG, LSS, LUSI, MILD, NELSON, NLST, UKLS) were included in the evidence synthesis [14,15,16,17,18,19,20,21,22,23,24,25,26,27,28,29,30,31,32,33,34,35,36,37,38,39,40,41,42,43,44,45,46,47,48,49,50,51,52,53,54,55,56]. One pilot study (UKLS) was not further investigated since it did not report results of the control group [24]. The 23 systematic reviews added no new studies but further citations on included RCTs. Eight RCTs reported results on mortality [16,17,18,19,20,21,22,23] and nine on lung cancer incidence [15,17,18,20,21,22,23,45,56]. One study (Depiscan) reported only baseline results [15], four on complications of follow-up procedures [20,33,37,41,46,55], five on the target or estimated effective dose per LDCT scan [22,27,35,36,40], and three on quality of life [25,29,34,48,51,53]. Overdiagnosis was formally assessed in only one study [14]. Detailed test characteristics for LDCT were reported for four studies [18,31,42,50]; numbers of false positives could be extracted or calculated for all studies.

### 3.2. Study Characteristics

Table 1 summarizes study characteristics and eligibility criteria of included RCTs. Six studies compared LDCT screening to no screening or usual care; three studies compared LDCT to CXR screening. The intervals between screening visits were usually one year, but two studies also included intervals of 2 and 2.5 years, respectively [16,43]. Between two and seven screening visits were scheduled, and duration of follow-up since randomization was between 1 and 10 years. Studies recruited men and women between 49 and 75 years of age with a smoking history of more than 20 pack-years. The DANTE study included only male participants, and the NELSON study only a small sample of female participants. In the considered RCTs, between 765 and 53,452 subjects were randomized, the average age of participants was 59.3 years, and most of them were male (56–100%; Table 2). Smoking history varied from an average of 32 to 54 pack-years and more current smokers were randomized to the screening arm than to the control arm except in the NLST study. Risk of bias was judged to be low in all but one study. The Italian MILD study revealed critical randomization issues [43] and is therefore only of moderate quality.

### 3.3. CT Scanning and Diagnostic Evaluation Algorithms Used in the Considered RCTs

In the considered RCTs, different CT scanners from four major manufacturers were used. With one exemption, they had less than or equal to 64 detector rows (Table 3). The acquisition protocols used a tube voltage between 80 and 120 kV and an effective tube current between 20 and 100 mA. Radiation exposure was reported as effective dose per LDCT scan that ranged between 0.4 to 2.4 mSv. In the ITALUNG and NLST study, organ doses for the lung were between 3.4 and 5.0 mGy and for the breast, between 3.0 and 4.9 mGy [35,36,40]. Four studies used volumetry software for a semi-automated estimation of the nodule size (DLCST, LUSI, MILD, NELSON).

### 3.4. Lung Cancer and Overall Mortality

**Figure 2:**
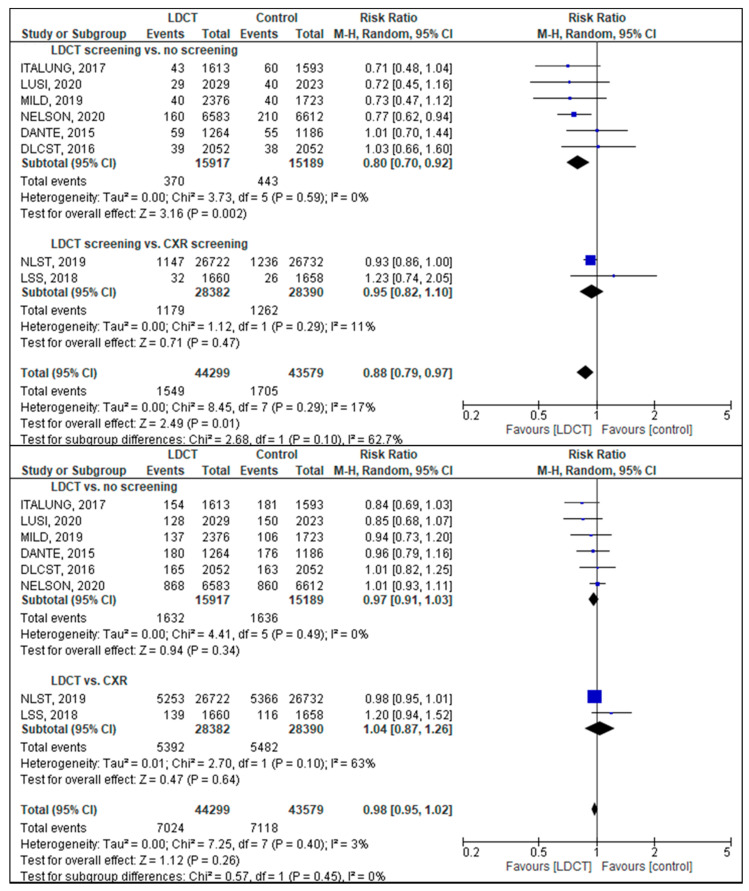
Forest plot for lung cancer mortality (**upper panel**) and overall mortality (**lower panel**). Each horizontal line presents an individual study with the square representing the effect estimate and the bars representing the 95% confidence interval. Studies are presented in subgroups according to the mode of control. The diamond on the bottom represents the pooled-effect estimate for subgroups and the overall analysis, respectively.

The analysis of lung cancer mortality comprised eight studies with data on 87,878 participants in the screening and control groups [16,17,18,19,20,21,22,23]. Overall, 1549 lung cancer deaths were observed among 44,299 screening participants and 1.705 lung cancer deaths among 43,579 controls in a follow-up period of 5.2 to 12.3 years. Only two studies were large enough to find statistically significant results [16,45]. Heterogeneity between studies in the main analysis was low (I^2^ = 17%) so that a pooled effect estimate could be calculated. The meta-analysis showed a statistically significant reduction of lung cancer mortality of 12% in the screening group as compared to the control group (risk ratio (RR) = 0.88; 95% confidence interval (CI): 0.79–0.97; Figure 2, upper panel).

The NLST study [19] contributed more than half of the subjects and therefore dominated the analysis. Exclusion of the study with moderate quality (MILD study) did thus not alter the result. A subgroup analysis excluding studies with CRX screening in the control arm showed a lung cancer mortality reduction higher than that of the main analysis (RR = 0.80; 95% CI: 0.70–0.92; Figure 2, upper panel). Although only three studies reported results stratified for gender, there seems to be a greater effect for women (women: RR = 0.71; 95% CI: 0.60–0.86; men: RR = 0.87; 95% CI: 0.77–0.97; data not shown) [16,23,45].

For all-cause mortality, most studies showed a tendency in favor of LDCT screening, but overall, no statistically significant difference between the study groups was found in the meta-analysis (RR = 0.98; 95% CI: 0.95–1.02; Figure 2 lower panel).

### 3.5. Lung Cancer Incidence

**Figure 3:**
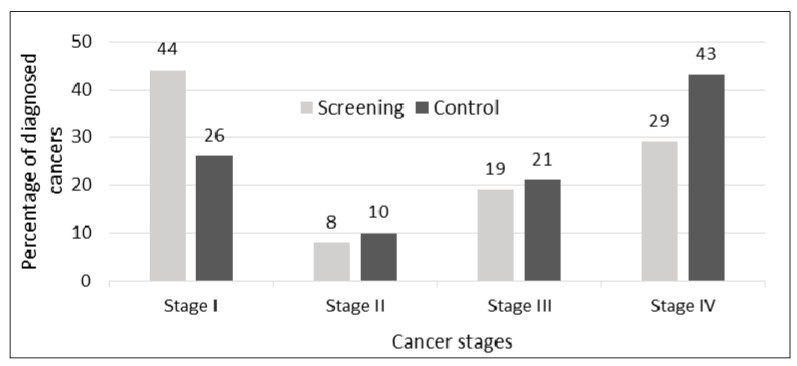
Distribution of diagnosed cancers by cancer stage and study group based on nine RCT.

The overall probability of getting a lung cancer diagnosis was 26% higher in the LDCT-screening group compared to the control group (Table 4; RR = 1.26; 95% CI: 1.10–1.45). The lung cancer detection rate at the first screening round (baseline) was 1.1% (SD: 0.5%) and decreased in the following screening rounds (Table 5). About one in five lung cancers in the screening group was non-screen-detected (mean: 22.4%, SD: 17.0%). Figure 3 gives the distribution of tumor stages of all diagnosed lung cancers in the screening and control groups. More cancers were detected in stage I in the screening group than in the control group (mean of all diagnoses: 44% vs. 26%) and less in stage IV (29% vs. 43%). The most frequent histologic type was adeno carcinoma, followed by squamous cell and small cell carcinomas (data not shown).

### 3.6. Consequences of Screening

A major problem of lung cancer screening is the high rate of false-positive test results and the associated risks of unnecessary diagnostic work-up. According to the discrepant definitions of screening results in the considered RCTs, between 3.6% (MILD annual screening) and 24.2% (NLST) of screening-LDCT scans were classified as indeterminate or positive (Table 5). The vast majority (84% to 96% [41,50]) of the positive scans turned out to be false positive, as they identified non-cancerous lesions. In the NELSON study, 67 out of 273 (24.5%) subjects with a false-positive result underwent a surgery or other invasive procedure for diagnostic work-up [32], and in the LUSI study, 90 out of 157 (57%) biopsies yielded benign lesions [23]. Complication rates associated with (invasive) work-up procedures were low (0.2–1.7%) [33,46].

Not all malignant lesions found by an LDCT screening would have caused symptoms or needed any treatment in a person’s lifetime. In a crude approach, this overdiagnosis was calculated as the excess in lung cancer incidence in the screening group [14]. Among studies with follow-up periods of at least four years after the last screening visit, the risk that a lung cancer diagnosis was an overdiagnosis ranged between 18.5% in the NLST and 69.1% in the DLCST study.

Three studies (DLCST, NELSON, NLST) assessed health-related quality of life and compared subjects in different study arms (screening versus control) and subjects with different screening results (negative, indeterminate, positive). Results from the DLCST indicate that participation in the screening trial might have little negative psychosocial consequences for persons in both the screening and the control arm [25,48]. There is some evidence that waiting for screening results caused psychological distress, particularly in subjects with indeterminate results [51,52,53]. Approximately 50% of the participants in the NELSON trial reported discomfort associated with waiting for the results of the LDCT scan [53]. However, if there was a negative effect of the screening situation, it was only transient and even statistically significant differences between groups were not clinically relevant [51,52,53].

Seven RCTs report that smoking cessation was offered in one or the other way. Some provided written information materials, whereas others offered counselling or participation in a cessation program [20,57,58,59,60,61,62,63,64,65,66,67,68,69]. One study (ITALUNG) combined behavioral and pharmacological interventions in a subgroup of participants who were still smoking after four years [61]. Written information and personal counselling reduced the intensity of smoking and the proportion of smokers equally in the screening and the control arms. Between 10% and 24% of smokers quit during the trials [57,59,62]. In the LUSI trial, for example, participants of both study arms were invited to attend a personal 15-min smoking cessation counselling with a psychologist [59]. The smoking prevalence decreased significantly by 4% in the entire cohort but did not differ significantly between study arms. The decline was more pronounced in the subgroup of attendees of the stop-smoking counselling and mounted up to 10%. Some studies investigated how the screening results affected smoking behavior (DLCST, NELSON, NLST) and what influence the individual motivation to stop smoking (DLCST) has on smoking cessation. They showed that a positive baseline LDCT scan as well as high individual motivation can significantly increase smoking cessation rates [57,58,65,66,68]. Overall, the studies showed heterogeneous results, and it was not possible to draw conclusions on the overall long-term changes in smoking behavior and on the effect that the screening participation might have.

## 4. Discussion

Based on a systematic literature review, we performed a meta-analysis of eight RCTs on LDCT lung cancer screening, including the latest results from European trials published in 2020, comprising a total of more than 87,000 participants. The estimated relative reduction of lung cancer mortality by screening with LDCT was 20% when compared to no screening and 12% across all considered studies, which confirms earlier studies [70,71,72,73]. Although the meta-analysis of included studies did not provide proof of a benefit in all-cause mortality, the effect estimates of most individual studies tend in the same direction as for the lung-cancer-specific mortality and indicate a protective effect.

The pooled result is mainly driven by the two largest studies, the U.S. NLST and the Dutch-Belgian NELSON trial. It may be criticized that the NLST used chest X-ray as comparator instead of no intervention; however, earlier studies suggested that X-ray lung screening has no significant effect and, therefore, that it could be treated as no screening [74]. We believe that the difference between the NLST lung-cancer-mortality results and the no-control subgroup of studies lies rather in the observation time than in the CRX control. The NLST mortality results in the meta-analysis include the extended follow-up with a median of 12.3 years. Active follow-up was performed only through 2009; thereafter, the subjects’ vital status was assessed by linkage with cancer registries. The authors noted that there might be a dilution of the screening effect with the risk ratio moving towards zero, when deaths from lung cancers that developed several years after the end of protocol screening are included. Their dilution-adjusted analysis showed a slightly greater effect on lung cancer mortality reduction (crude RR = 0.92; 95% CI: 0.85–1.00 vs. dilution-adjusted RR = 0.89; 95% CI: 0.80–0.997) [19], which was still smaller than the earlier results after 6.5 years follow-up (RR = 0.84; 95% CI: 0.75–0.95) [45]. These differences emphasize the relevance of the duration of the follow-up and the limitations for extrapolation of results from studies with a limited number of study rounds to the population-screening setting.

As expected, more lung cancers were detected in the screening group, and these tumors were more likely to be in an early stage. But these diagnoses include also indolent cancers that would not progress to a clinically manifest disease. The fraction of overdiagnosis among screen-detected cancers can only be estimated. A meta-analysis by Brodersen et al. included five RCTs on lung cancer screening by LDCT and found a risk of overdiagnosis of 38% [75], which is within the range of studies considered in this review. Estimates calculated as the excess incidence in the screening group may overestimate the real problem, as incidence rates in the control group might catch up on a longer perspective [19]. Overdiagnosis in trials is strongly dependent on the duration of follow-up, the individual remaining life expectancy, and competing risks of death. Modelling studies that include these parameters report a risk of overdiagnosis of 8 to 14% of screen-detected cases and emphasize that it is a major issue particularly in older participants due to competing causes of death [76].

False-positive LDCT results were common and led to invasive procedures for benign lesions. The definition of a positive LDCT scan and the management of nodules is crucial for screening effectiveness, in particular when work-up involves invasive procedures with risk of complications. So far, there is no agreement on the optimal cut-off size for nodules classified as positive and on the best management of indeterminate findings. Several studies applied volumetric measurement and used the volume doubling time of a nodule as an indicator for malignancy. Analyses of NELSON trial data indicate that a higher accuracy and reproducibility of LDCT reading can be achieved by semi-automatic volume measurement [77]. But even with refined evaluation of nodules, many individuals need follow-up visits and interventions. In the LUSI trial, for example, about 22% of screened subjects were recalled after the baseline scan, 2.6% underwent biopsy, and only 1.1% had lung cancer diagnosis confirmed. The probability of a person not having lung cancer after a negative LDCT result was close to 1 in most studies.

There is consensus that only high-risk populations should be screened, but the best definition of this group is yet unclear. The aim is to select individuals in a manner that maximizes the reduction of lung cancer mortality and minimizes false-positive test results that induce biopsies or surgeries for benign lesions, overdiagnosis, over-therapy, and radiation risk. Most RCTs applied a small set of simple inclusion criteria, like age, pack-years, and years since quitting, with slight variations. Modeling studies for the U.S. Preventive Services Task Force have shown that annual screening from age 55 through 80 for ever-smokers with at least 30 pack-years and ex-smokers with less than 15 years since quitting was the most advantageous screening strategy [76]. Evidence is emerging that an individual risk-based approach might be more effective [78,79]. A number of well-calibrated risk-models, such as the PLCO_M2012_ model, exist and could be adapted to national populations [80]. There seems to be also a possibility to individualize the interval between screening visits. The studies assessed in this review had between two and five screening rounds with intervals of 1, 2, or 2.5 years, respectively. The NELSON study demonstrated that a 2.5-year interval reduced the effect of screening and resulted in more interval cancers and more advanced tumors than a one-year or a two-year interval [56]. However, it also yielded that subjects with a negative baseline LDCT scan had a low probability to have a positive scan one year later, so it may be speculated that a biennial screening regimen might be efficient for selected participants and reduce radiation exposure [32,81].

There is no general definition of a low-dose CT scan, and the effective doses of a single scan varied in the considered studies from 0.4 mSv to 2.4 mSv. Over the last few years, rapid innovations of CT technology have not only improved image quality and thus the diagnostic accuracy but also markedly reduced the radiation dose per scan. Nevertheless, the cumulative radiation dose from repeated LDCTs cannot be neglected from a radiation-hygienic point of view. This aspect will be treated in detail in a subsequent publication in order to assess the benefit-risk-ratio of various screening scenarios.

Participation in a lung cancer screening can have the potential to change quality of life in both directions. On the one hand, indeterminate results requiring a follow-up examination after a few month may cause psychological distress, whereas negative scans may reassure the participant. The studies considered here did not show a clear effect of screening on health-related quality of life and psychosocial consequences.

Primary prevention is the best way to reduce lung cancer risk [82]. According to the included studies, it is questionable whether participation in screening alone can influence changes in smoking behavior. Therefore, lung cancer screening should be coupled with the offer of those smoking cessation interventions that have been shown to be effective [83].

For implementation of LDCT screening on a population level, it must be noted that the benefit of screening was shown in RCTs with standardized protocols and high-quality demands. Even then, negative consequences of screening, like unnecessary biopsies and surgeries, will affect large numbers of individuals. Extrapolation of the NLST results to a nationwide screening program in Germany suggests about 12,500 complications during three years of screening [84]. Therefore, efforts should be made to install measures that assure quality of the entire screening process, including the training and education of personnel, the required equipment, performance of the examination, image reading, the type and scope of diagnostic workup, and the documentation [85].

## 5. Conclusions

The meta-analysis of RCTs on LDCT lung cancer screening presented in this review takes into account the most recent results of two European screening studies published in 2020. It demonstrates a favorable effect of LDCT screening on lung cancer mortality. The results presented will form the evidence base for the decision regarding the regulatory approval of LDCT lung cancer screening in Germany. To translate the benefit of screening as shown by the considered RCTs to a population-based screening activity, high-quality standards and stringent requirements, as in the RCTs, have to be implemented. To meet these standards, the screening activities should be embedded in a structured screening process and involve interdisciplinary medical teams with expertise in radiology, pulmonology, and thoracic surgery.

## Figures and Tables

**Table 1:** Study characteristics, eligibility criteria, and intervention.

Study Country, Recruitment Period	Study Sites	Age Range (Years)	Smoking History	Smoking Abstinence (Years)	Screening Interval (Years)	Screening Visits	Control Group	Follow-Up (Years)
DANTE Italy, 2001–2006	3	60–74	≥20 PY	<10	1	5	no screening	8.35
DEPISCAN France, 2002–2004	14	50–75	≥15 cig/day for 20 yrs	<15	1	3	CXR	not reported
DLCST Denmark, 2004–2006	1	50–70	≥20 PY	<10	1	5	no screening	9.8
ITALUNG Italy, 2004–2006	3	55–69	≥20 PY		1	4	no screening	9.3
LSS USA, 2000	6	55–74	≥30 PY	<10	1	2	CXR	1
LUSI Germany, 2007–2011	1	50–69	≥15 cig/day for 25 yrs OR ≥10 cig/day for 30 yrs	<10	1	5	no screening	8.9
MILD Italy, 2005–2011	1	49–75	≥20 PY	<10	1/2	7/4	no screening	10
NELSON Belgium/The Netherlands, 2003–2005	4	50–75	≥15 cig/day for ≥25 yrs OR ≥10 cig/day for ≥30 yrs	<10	1/2/2.5	4	no screening	8.16
NLST USA, 2002–2004	33	55–75	≥30 PY	<15	1	3	CXR	12.3

cig, cigarettes; CXR, chest radiogram; PY, pack-years; yrs, years.

**Table 2:** Characteristics of participants.

	Subjects Randomized	Subjects in Screening Arm	Male Participants (%)	Age (Years), Mean or Median	Pack-Years, Mean or Median	Current Smokers in Screening vs. Control Group (%)
DANTE	2450	1264	100	64	47	56.5 vs. 57.4
DEPISCAN	765	385	71	56	32	65 vs. 64
DLCST	4104	2052	56	58	36	75.3 vs. 76.9
ITALUNG	3206	1613	64.7	61	40	66.5 vs. 63.1
LSS	3318	1660	59	68% younger than 64	54	57.9 vs. 57.1
LUSI	4052	2029	64.7	55	-	50.2 vs. 49.8
MILD	4099	annual: 1190 biennial: 1186	68.4	58	39	68.9 vs. 89.7
NELSON	15,822	7915	84	59	42	55.5 vs. 54.8
NLST	53,452	26,722	59	43% younger than 59; 26% older than 65	-	48.1 vs. 48.1

**Table 3:** Technical parameters, radiation dose, and criteria for follow-up procedures.

	CT Detector Rows	Use of Volumetry Software	Tube Voltage (kV)	Effective Tube Current (mA) or Current-Time-Product (mAs)	Effective Dose Per LDCT Scan (mSv)	Minimal Nodule Size or Growth for Recall/Follow-Up
DANTE	1–16	no	140	40 mA	n. r.	All solid, non-smooth
DEPISCAN	n. r.	no	100–140	20–100 mA	n. r.	D > 5 mm
DLCST	16	yes	120	40 mA	around 1	D ≥ 5 mm
ITALUNG	1–64	no	120–140	20–43 mA	1.2–1.4	D > 5 mm
LSS	n. r.	no	120–140	60 mA	n. r.	Baseline: D > 3 mm; other rounds: D > 4 mm
LUSI	16 and 128	yes	n. r.	n. r.	< 1.6–2.0	D ≥ 5 mm or VDT = 400–600 days and D < 7.5 mm
MILD	16	yes	120	30 mAs	n. r.	V ≥ 60 mm^3^ or D ≥ 5 mm
NELSON	16 and 64	yes	80–140	n. r.	0.4–1.6	D > 5 mm or V > 50 mm^3^ or VDT = 400–600 days
NLST	4–64	no	120	20–40 mAs	1.6–2.4	D > 4 mm

n. r., not reported; D, diameter; V, volume; VDT, volume doubling time.

**Table 4:** Lung cancer incidence and mode of detection.

	Number of Diagnosed Lung Cancers (%)		Number of Non-Screening Detected Lung Cancers in the Screening Group (%)
	Screening	Control	
DANTE	104 (8.2)	72 (6.1)	38 (37)
DEPISCAN	8 (2.1)	1 (0.3)	not reported
DLCST	100 (4.9)	53 (2.6)	not reported
ITALUNG	67 (4.2)	71 (4.4)	25 (37)
LSS	40 (2.4)	20 (1.2)	2 (5)
LUSI	85 (4.2)	67 (3.3)	6 (7)
MILD	98 (4.1)	60 (3.5)	27 (28)
NELSON	344 (4.3)	304 (3.8)	141 (41)
NLST	1701 (6.4)	1681 (6.3)	44 (3)

**Table 5:** Performance characteristics of LDCT as screening test.

	Number of Screening-LDCTs Performed	Positive or Indeterminate LDCT Findings N (%)	Recall Rate (%)	Lung Cancer Detection Rate (%)
DANTE	6482	-	Tt: 28.1	Tt: 5.3
DEPISCAN	336	81 (24.1)	Tt: 24	Tt: 2.4
DLCST	9800	512 (5.2)	T0: 7.6	T0: 0.83
				Tt: 0.70
ITALUNG	5333	1044 (19.6)	T0: 30.3	T0: 1.5
			Tt: 52.7	Tt: 0.5
LSS	2984	655 (22.0)	T0: 25.8	T0: 1.9
			Tt: 34.5	T1: 0.57
LUSI	9405	Positive: 174 (1.9)	T0: 22.2	T0: 1.1
		Indeterminate: 642 (6.8)		
MILD				
annual	7369	Positive: 91 (1.2)	T0: 14.8	T0: 0.96
		Indeterminate: 177 (2.4)	Tt: 5.81	Tt: 0.56
biennial	5006	Positive: 59 (1.2)	T0: 13.7	T0: 0.52
		Indeterminate: 158 (3.1)	Tt: 6.97	Tt: 0.56
NELSON *	22,600	Positive: 467 (2.1)	T0: 20.4	T0: 0.9
		Indeterminate: 2069 (9.2)		Tt: 3.2
NLST	75,126	18,146 (24.2)	T0: 27.3	T0: 1.1
			Tt: 24.2	

* NELSON: only male participants. T0, first screening round (baseline); Tt, all screening rounds.

## Data Availability

Data sharing not applicable.
